# Medical student plagiarism in problem-based learning courses

**DOI:** 10.3402/meo.v21.30537

**Published:** 2016-01-11

**Authors:** Kyong-Jee Kim, Jee Young Hwang, Dong-Wook Lee, Min-Sung Shim

**Affiliations:** 1 Department of Medical Education, School of Medicine, Dongguk University, Gyeongju, South Korea; 2 Department of Family Medicine, School of Medicine, Dongguk University, Gyeongju, South Korea

Students’ academic misconduct has been an issue in medical education and is more likely with development of technology (1, 2). We investigated the occurrence of plagiarism by medical students in a problem-based learning (PBL) course. The participants were a cohort of Year 1 students in the 4-year medical program (*n*=53) at Dongguk University Medical School in South Korea. Of these students, 38.5% were female and 61.5% were male. Of these, 60% were graduate-entry students and 40% were undergraduate-entry students. Student ages ranged from 19 to 33 years (*M*=24.13, SD=3.19).

The students turned in papers after self-study of topics on the PBL module. The plagiarism detection program offered by the university was used for the investigation. Thirty-three students (62%) plagiarized, mainly copying and pasting websites found using Google, a Korean search engine, or the one offered by a Korean medical center. As a result of such extensive use of limited resources and searching the Internet using similar keywords, contents of the papers were very similar. In another assignment, students wrote their reflections on ethical issues raised in the module. Seventeen students (32%) plagiarized papers written by their peers; some of them copied and pasted others’ work and in some cases they used ideas from what their peers had written.

In addition, we conducted one-on-one interviews with all of the students who were found to have plagiarized to investigate their patterns and perceptions of plagiarism. We found that most of the students were not aware that copying information from the website without proper citation of sources was considered plagiarism, and they were not aware that copying reports of their peers was a serious problem. In addition, most of the students copied papers written by their peers who were neither in their social network nor in the same PBL group.

Our study indicates that plagiarism in PBL is as prevalent as in other conventional courses and that its occurrence differs according to the type of assignment. In addition, all students should be monitored across PBL groups for detection of plagiarism because they likely copy papers written by peers in other PBL groups or those outside of their social networks. In conclusion, it is suggested that students should be educated on plagiarism to enhance their awareness of what it is and how to avoid it. A variety of educational interventions may be available to teach about plagiarism to medical students – from conventional lectures to online tutorials. In addition, students need to be offered various learning resources for their self-study in order to prevent student plagiarism. Offering diverse, quality learning resources is fundamental to fostering an effective learning environment for PBL (3), and this can also encourage students to use diverse resources instead of merely copying and pasting content from simple Internet search in writing up papers.

*Kyong-Jee Kim* Department of Medical Education School of Medicine, Dongguk University Gyeongju, South Korea Email: kjkim@dongguk.ac.kr*Jee Young Hwang* Department of Medical Education School of Medicine, Dongguk University Gyeongju, South Korea*Dong-Wook Lee* Department of Family Medicine School of Medicine, Dongguk University Gyeongju, South Korea*Min-Sung Shim* Department of Family Medicine School of Medicine, Dongguk University Gyeongju, South Korea
